# Lipid-coated albumin-paclitaxel nanoparticles loaded with sorcin-siRNA reverse cancer chemoresistance via restoring intracellular calcium ion homeostasis

**DOI:** 10.1186/s12951-022-01487-6

**Published:** 2022-07-07

**Authors:** Chenglong Wang, Xiaolin Xu, Peipei Zhang, Shuhan Xiong, Jia Yuan, Xuzhu Gao, Wencai Guan, Fanchen Wang, Xin Li, Hongjing Dou, Guoxiong Xu

**Affiliations:** 1 Research Center for Clinical Medicine, Jinshan Hospital, Fudan University, Shanghai, 201508 People’s Republic of China; 2 State Key Laboratory of Metal Matrix Composites, School of Materials Science and Engineering, Shanghai Jiao Tong University, Shanghai, 200240 People’s Republic of China

**Keywords:** Drug resistance, Malignant tumor, Nanoparticle, Reversal, TGF-β signaling

## Abstract

Chemoresistance is often a cause of the failure of chemotherapy in cancer treatment. Sorcin (SRI) is a soluble resistance-related calcium-binding protein involved in chemoresistant processes and is overexpressed in many chemoresistant cancer cells, including paclitaxel (PTX)-resistant ovarian cancer. Increased SRI can reduce the concentration of calcium ions in the cytosol and mitochondria and the decrease of calcium ion concentration prevents the occurrence of apoptosis. Here we examined the SRI expression in multiple cancers using a human TissueArray and found that SRI expression was significantly higher in malignant tumor tissues. Furthermore, SRI was overexpressed, while intracellular calcium concentration was decreased, in chemoresistant cancer cells. To restore intracellular calcium homeostasis and overcome chemoresistance, we developed lipid-coated albumin-PTX nanoparticles loaded with SRI-siRNA (LANP-PTX-siSRI) for PTX and SRI-siRNA co-delivery. LANP-PTX-siSRI had dual-target roles in the regulation of SRI and the delivery of PTX into chemoresistant cells. The LANP-PTX-siSRI inhibited the expression of SRI and enhanced intracellular calcium, leading to the induction of apoptosis and the inhibition of the growth of PTX-resistant cancer cells in vitro and in vivo. In addition, the mechanism study revealed that the overexpression of SRI was associated with an impaired TGF-β signaling pathway. The administration of TGF-β1 inhibited two calcium-binding proteins SRI and S100A14. In conclusion, our data unveil that restoring intracellular calcium ion homeostasis via reducing SRI expression can reverse chemoresistance. Thus, the fabricated LANP-PTX-siSRI has a potentially therapeutical application.

**Supplementary Information:**

The online version contains supplementary material available at 10.1186/s12951-022-01487-6.

## Introduction

Chemotherapy remains the main application for the treatment of patients with advanced cancer [1]. However, patients often develop chemoresistance during chemo-drug administration. Hence, chemoresistance has been recognized as the main cause of tumor chemotherapy failure [2]. Paclitaxel (PTX) in combination with platinum has long been the recommended chemotherapy regimen for cancer treatment [3]. PTX is a classic anti-mitotic drug and its cytotoxic effect mainly depends on increasing assembly and preventing depolymerization of microtubules [4, 5], thus causing the stop of cancer cell proliferation and the induction of apoptosis. Most cancer patients have an initial effect dring chemotherapy, and yet they often develop chemoresistance and eventually recur after a period of treatment [6, 7]. The mechanisms of cancer chemoresistance include, but do not limit, the enhancement of the repair of drug-induced DNA damage, increase of intracellular drug efflux, and inhibition of apoptosis induction [8, 9].

Sorcin (SRI) is a soluble resistance-related calcium-binding protein, which is overexpressed in many malignant tumors [10]. SRI as a calcium buffer can balance the level of calcium concentration by either actively pumping calcium ions into the extracellular space from the cytoplasm or flowing into the endoplasmic reticulum and mitochondria [11], thus regulating calcium homeostasis within a cell [12]. SRI can not only bind calcium ions but also can interact with the calcium channel proteins and regulate the sodium-calcium pump located in the endoplasmic reticulum and serosal membrane [13]. An abnormal expression of SRI has been found in chemoresistant cells [14, 15]. The increased expression of SRI can induce the accumulation of calcium ions in the endoplasmic reticulum and reduce the concentration of calcium ions in the cytosol and mitochondria [16]. The decrease of calcium ion concentration prevents the occurrence of apoptosis in the mitochondrial apoptosis pathway [17, 18]. SRI can drive the chemoresistance process [19] and malignant progression by regulating the function of P-glycoprotein (P-gp) encoded by the ATP binding cassette subfamily B member 1 (*ABCB1*) gene in multidrug-resistant cancer cells [20]. P-gp is an important drug-resistant protein that pumps chemo-chemicals out of cells [21], thus enabling cancer cells resisting to a drug.

In recent years, siRNA has made great progress in tumor treatment [22]. siRNA can inhibit target gene expression by binding with specific mRNA transcripts [23]. However, siRNA is limited in clinical application due to its nature, such as low internalization in tumor cells, degradation by enzymes in the bloodstream, off-targeting features, and so on [24]. How to successfully transport siRNA to tumor cells is a bottleneck for the application of siRNA in clinical practice. siRNA can be effectively protected from nuclease degradation by nano-carrier loading and can be enriched in the tumor by the enhanced permeability and retention (EPR) effect, and further, nano-carrier loading can also diminish immune stimulation [25].

**Scheme 1:**
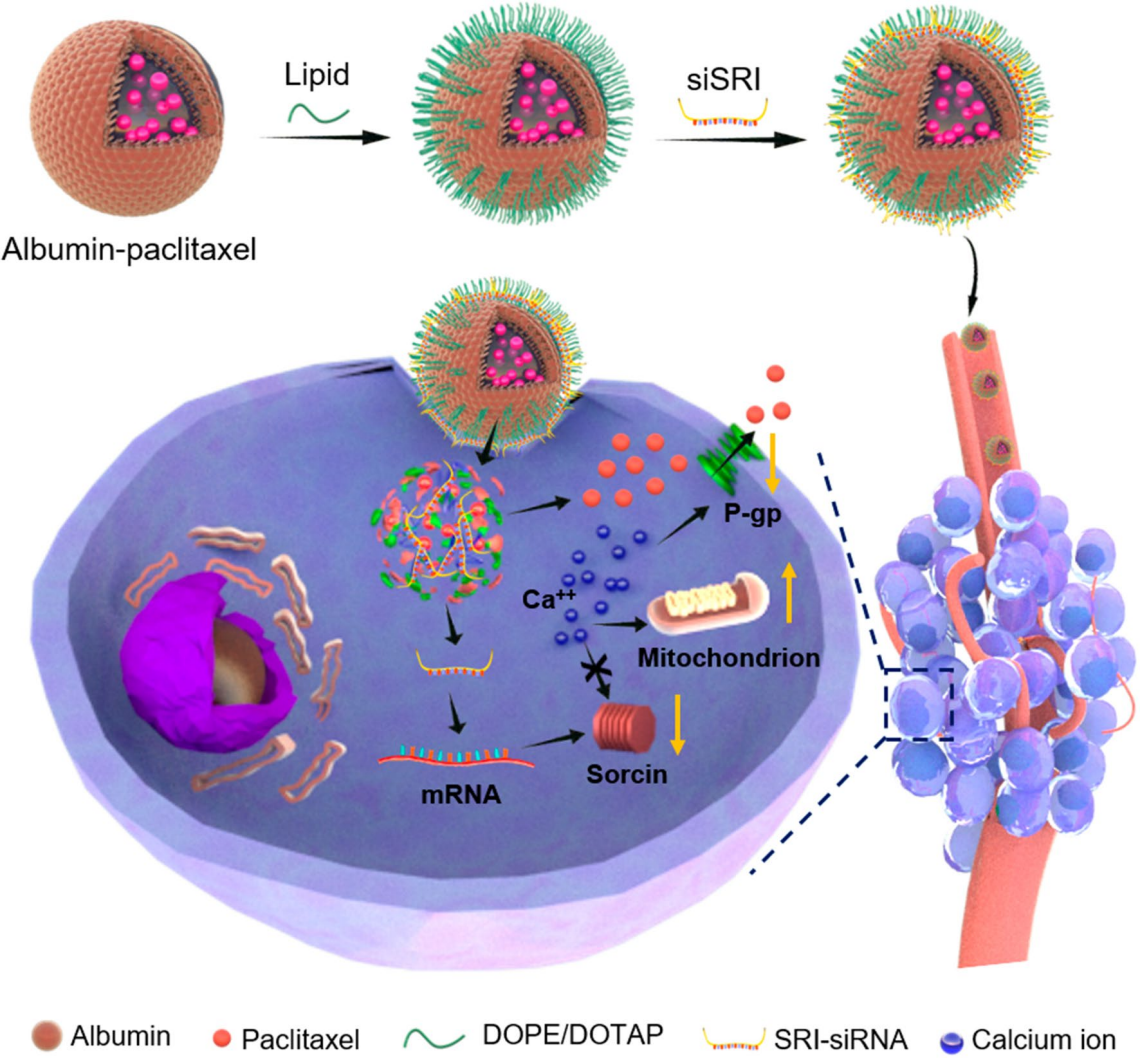
Schematic illustrations of the construction of lipid-coated albumin nanoparticles with PTX and SRI-siRNA (LANP-PTX-siSRI) and the depiction of LANP-PTX-siSRI work on revising chemoresistance. Lipid consists of DOPE/DOTAP. Ca^++^, calcium ions; DOPE, dioleoylphosphatidylethanolamine; DOTAP, 1,2-dioleoyl-3-trimethyl-ammonium-propane; P-gp, glycoprotein; SRI, sorcin; siSRI, SRI-siRNA

To reduce the expression of SRI in chemoresistant cancer cells, we developed novel lipid-coated albumin-PTX nanoparticles for PTX and SRI-siRNA co-delivery (LANP-PTX-siSRI) that inhibited the expression of SRI and enhanced intracellular calcium, leading to the induction of apoptosis and the inhibition of PTX-resistant cancer cell growth. As shown in Scheme 1, siSRI can bind to the mRNA to reduce the expression of SRI, whereas PTX can induce the apoptosis of chemoresistant cancer cells, thus reversing the chemoresistance of cancer cells.

## Materials and methods

### Cell lines

PTX-resistant ovarian cancer cell line A2780/PTX and its sensitive counterpart A2780 were purchased from Keygen Biotech (Nanjing, Jiangsu, China). PTX-resistant lung carcinoma cancer cell line A549/PTX was purchased from Keygen Biotech and PTX sensitive cell line A549 was purchased from American Type Culture Collection (ATCC, Manassas, VA, USA). A2780/PTX and A2780 were cultured in DMEM, while A549/PTX and A549 were cultured in RPMI-1640 medium (Gibco, Invitrogen, Carlsbad, CA, USA), supplemented with 10% FBS (Invitrogen).

### Tissue microarray and immunohistochemical analysis of multiple organs

The tissue microarray was obtained from Shanghai Outdo Biotech Company (Cat# HOrgC120PG05, Shanghai, China). The ethical approval of using human multiple organs was approved by the Ethics Committee of Shanghai Outdo Biotech Company (No. SHYJS-CP-1904010). Immunohistochemical analysis was performed in Shanghai Outdo Biotech Company (Shanghai, China). The immunohistochemical tissue microarray was imaged by using an Aperio ImageScope system (v12.4.35008).

### Construction and characterization of LANP-PTX-siSRI

The lipid modification method was the same as the previous report [26]. In brief, 50 mg of DOPE and 50 mg of DOTAP were dissolved in 4 mL of chloroform. The solvent was then moved by rotary evaporation at room temperature. Lipid suspension was obtained by hydration with 5 mL ultrapure water, followed by mixing with 20 mg/mL of albumin-PTX (Jiangsu Hengrui Pharmaceuticals Co., Ltd., Jiangsu, China) at a weight ratio of 1:1. The lipid-coated albumin-PTX nanoparticles (LANP-PTX) were obtained after ultrasonic dispersion and 1% (w/w) dextran nanogels, as described in the previous report [26], were added to reduce precipitation generation. LANP-PTX-siSRI and LANP-PTX-siNC were prepared by mixing LANP-PTX (16 mg/mL) either with siSRI (20 μM) or with siNC (20 μM) in an equal volume and vortexing for 20 s at room temperature. The control LANP (without PTX) was prepared by mixing 20 mg/mL of albumin (Sigma-Aldrich, USA) with lipid suspension at a weight ratio of 1:1 and then, underwent ultrasonic dispersion. LANP-siSRI and LANP-siNC were prepared by mixing LANP (16 mg/mL) either with siSRI (20 μM) or siNC (20 μM) in an equal volume and vortexing for 20 s at room temperature.

Diameter and Zeta potential were measured using a Zetasizer Nano instrument (ZS90, Malvern, U.K.). Images were recorded using a transmission electron microscope (TEM, 120 kV, FEI Tecnai G2 Spirit BioTwin, FEI, U.S.A.).

### Release of siRNA

A 250 µL of LANP-PTX-siSRI (16 mg/mL) was added to a microdialysis tube (4000 MW, Sangon Biotech, Shanghai, China). Then, the microdialysis tube was put into a microdialysis cup (Sangon Biotechnology, Shanghai, China) with 25 mL of pure water. The dialysate was taken at various times and the siRNA release was determined by measuring siRNA concentration in dialysates using Nanodrop (Thermo Fisher, USA).

### Flow cytometry analysis

The cells were cultured in 6-well plates at a density of 2 × 10^5^ cells/well for 24 h. Then, cells were trypsinized, centrifuged, and stained with a calcium ion assay kit (Beyotime Biotechnology, Shanghai, China) according to the product instructions. The cells were resuspended with 500 µL PBS and detected by flow cytometry (Gallios, Beckman Coulter, CA, USA).

A 5 μL of Fam-siRNA (20 μM) was loaded on 10 μL of LANP (8 mg/mL) and added to 6-well plates. After 4 h, cells were trypsinized, centrifuged, and resuspended with 500 µL PBS, and then detected by flow cytometry. Flow cytometry data were analyzed using FlowJo (V10).

### Laser confocal microscopy assays

The cells were cultured in a confocal petri dish with a 35 mm diameter and 20 mm glass bottom. After the confluence reached 60–80%, cells were stained with a calcium ion assay kit according to the product instructions. The nuclei were further stained with Hoechst 33,342 (Beyotime Biotechnology, Shanghai, China). After washing with PBS 3 times, cells were photographed by a laser confocal microscope (SP8 STED 3X, Leica, Germany).

### AGE assay

Green fluorescent Fam-labeled siRNA (20 μM in 2.5 μL, Fam-siRNA) was loaded into 5 μL of LANP (8 mg/mL). Electrophoretic separation was performed using a 2.5% (w/v) agarose gel containing SYBR Green Nucleic Acid Gel Stain. Electrophoresis was conducted in tris–acetate (TAE) running buffer at a voltage of 140 V for 20 min. Then, the gel was visualized and recorded using ultraviolet transmittance (Bio-Rad GelDoc EZ, USA).

### RNA extraction and qRT-PCR

Total RNA was extracted using an RNA Rapid Purification kit (ES Science, Shanghai, China) according to the manufacturer’s instructions. PCR was performed using a qPCR reverse transcription kit (Mei5 Biotechnology, Beijing, China). The sequence of PCR primers was shown in Additional file 1: Table S1. A 7300 real-time PCR system (V1.4, Applied Biosystems, USA) was used to measure threshold circulation (Ct) and target gene expression was normalized to endogenous gene GAPDH.

### RNA sequencing analysis

Total RNA was extracted using an RNA Rapid Purification kit (ES Science, Shanghai, China) according to the manufacturer's instructions. mRNA sequencing was conducted by Shanghai Biochip Co. Ltd. (Shanghai, China).

### Protein extraction and Western blot

Cells were lysed with a sodium dodecyl sulfate lysate (SDS) containing benzyl sulfonyl fluoride (1%, w/v) and phosphatase inhibitor (1%, w/v). After ultrasonic lysis, the total protein was subjected to SDS–polyacrylamide gel electrophoresis. The primary antibodies were rabbit anti-P-gp (1:5000 diluent, CST, USA), rabbit anti-SRI (1:1000 diluent, CST, USA), mouse Smad2 (1:1000 diluent, CST, USA), rabbit P-Smad2 (1:1000 diluent, CST, USA), and mouse anti-β-actin (1:5000 diluent, CST, USA). The secondary antibodies were goat anti-rabbit IgG (1:10,000 diluent, Proteintech, USA) and anti-mouse IgG (1:10,000 diluent, Proteintech, USA) labeled with horseradish peroxidase. A chemiluminescence imaging system (Tanon Science & Technology, China) was used to photograph protein bands.

### Cell viability and cell toxicity

Cells were seeded into 96-well plates at a density of 1 × 10^4^. After 24 h, the medium was replaced by the fresh medium containing different concentrations of PTX ranging from 0.001–0.313 μM for sensitive cells and 0.625–40 μM for resistant cells. Each sample was performed with at least 4 parallel repetitions. After 48 h, a CCK-8 assay (Dojindo, Japan) was used to detect cell cytotoxicity by measuring the optical density (OD) of each well at 450 nm. The cell cytotoxicity calculation formula was used as indicated: cell cytotoxicity rate (%) = OD test /OD control × 100%.

### In vitro therapy

The cells were cultured into 96-well plates at a density of 1 × 10^4^/well. After cultivation for 24 h, the supernatant was removed and a fresh medium containing LANP-PTX, siRNA control (LANP-PTX-siNC), or LANP-PTX-siSRI was added to each well. The concentration of PTX was 0–20 μM and the corresponding siRNA concentration is in the range of 0–0.43 μM. After 48 h, cell viability was detected by CCK-8.

### RBC hemolysis study

A hemolysis test was performed by using rabbit blood. The 5% erythrocyte suspension was mixed with a four-time volume of LANP-PTX-siSRI solution (test group), PBS (negative group), and water (control), respectively. After incubation at 37 ℃ for 4 h, the supernatant was collected by centrifugation. The optical density (OD) of the supernatant was measured at 577 nm. The hemolysis rate was calculated by the following formula: Hemolysis rate (%) = (OD_test_—OD_negative_)/(OD_control_—OD_negative_) × 100%.

### Generation of tumor-bearing mice

The ethics approval of animal experiments was approved by the Ethics Committee of Shanghai Public Health Clinical Center (No. 2020-A027-01). About 3–4 weeks old female BALB/c nude mice (B&K Laboratory Animal Co., LTD., Shanghai, China) were raised under standard feeding conditions with adequate food and water. After 3 days, 3 × 10^6^ A549/PTX cells in 100 µL of RPMI-1640 medium without FBS were injected into the right side of each mouse. After 3 weeks, the tumor-bearing mice were randomly assigned to various treatment groups.

### Fluorescence imaging of tumor-bearing mice

Fluorescent-labeled LANP were fabricated by replacing DOPE with DOPE-Cy5.5 during the LANP preparation process. DOPE-Cy5.5 was synthesized as described previously [26]. The mice were intravenous by 100 μL of Cy5.5-labeled LANP solution with 0.25 mg of PTX and 6.25 nM of SRI-siRNA. In vivo fluorescence imaging was performed at 0.5, 1.5, and 5 h after intravenous injection. The ex vivo fluorescence of major organs and tumors was imaged.

### In vivo therapy

The tumor-bearing mice were divided into 3 groups (n = 8 in each group), including the normal saline group, the albumin-PTX group, and the LANP-PTX-siSRI group. The mice were intravenous and intratumor administration once every 2 days a total of 4 times. The corresponding solution is 100 μL (50 μL of intravenous + 50 μL of intratumor) each time. The dose of PTX was maintained at 12.5 mg/kg and the dose of siRNA was maintained at 312.5 nM/kg. The tumor volume and body weight were monitored every 2 days for 2 weeks. Tumor volume was calculated by *V* = *ab*^*2*^*/2*, where *V* is the tumor volume, *a* is the length of the tumor, and *b* is the width of the tumor. At the end of the experiment, the mice were anesthetized and sacrificed. The excised tumors were weighed and photographed.

### Pathological assessment

The tumors harvested from various treated groups were fixed with formalin and embedded in paraffin. A pathological examination was performed by Shenghua Biological Technology Co., LTD (Shanghai, China). Tumor tissue sections were stained with H&E, PCNA, and TUNEL and were photographed under a microscope.

### TGF-β treatment

TGF-β treatment was carried out by seeding the cells into a 6-well plate at a density of 2 × 10^5^ cells/well. After incubation for 24 h, a fresh medium containing TGF-β1 (R&D Systems, MN, USA) was added to wells at concentrations of TGF-β1 at 0, 1, and 10 ng/mL. The cells were collected for subsequent experiments after 48 h.

### Bioinformatics analysis

UCSC XENA (https://xenabrowser.net/datapages/) was used for the analysis of SRI expression levels in human serous ovarian cancer and lung adenocarcinoma. Data of lung adenocarcinoma and serous ovarian cancer were extracted from TCGA, and corresponding normal tissue data were extracted from GTEx. The prognostic value of SRI was assessed by Kaplan–Meier Plotter (www.kmplot.com) as described in our previous report [27].

### Statistical analysis

The qRT-PCR data are presented as mean ± standard error (SE). The cell viability, IC_50_, particle size, Zeta potential, tumor volume, tumor weight, and mice body weight are presented as the mean ± standard deviation (SD). Results were performed for assays at least 3 times. Graphs were drawn using Origin (V5.0, OriginLab, USA) or GraphPad (V5.0, GraphPad Software, USA) software. The student's *t*-test was used for statistical analysis of differences between the 2 groups. A p < 0.05 was considered statistically significant.

## Results and discussion

### Sorcin is overexpressed in malignant tumors

**Fig. 1:**
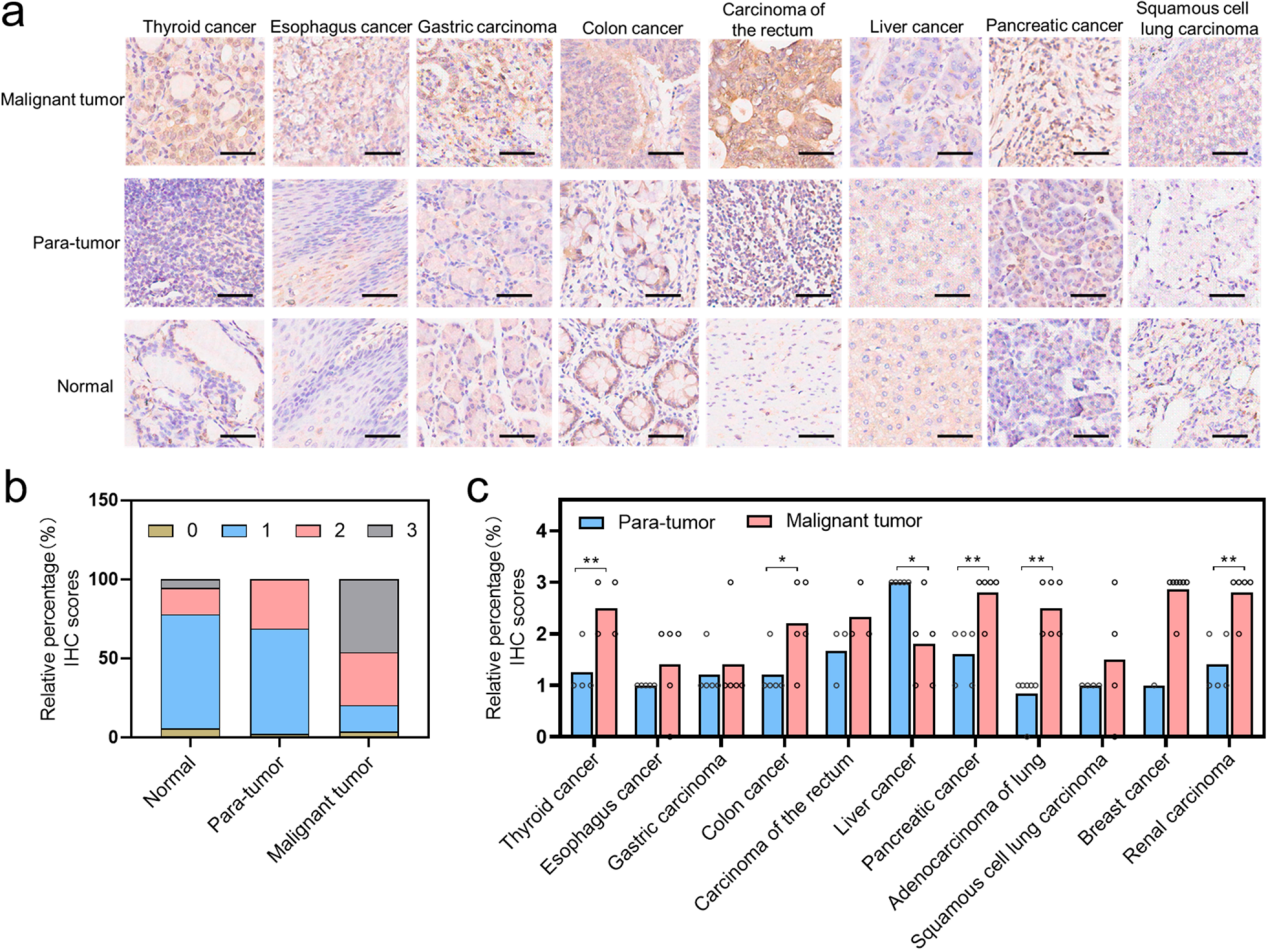
SRI protein expression in a human tissue microarray detected by immunohistochemistry (IHC). **a** Representative images of SRI protein expression in malignant tumor tissues, para-tumor tissues, and normal tissues. Scale bar, 50 μm. **b** Comparison of SRI protein expression between human malignant tumor tissues (n = 54), para-tumor tissues (n = 48), and normal tissues (n = 18). IHC scores of 0, 1, 2, and 3 represent negative, weak-positive, moderate-positive, and strong-positive, respectively. **c** Comparison of SRI protein expression between human malignant tumor tissues and para-tumor tissues in human different cancers. One circle represents one tissue sample. *P < 0.05; **P < 0.01

Our previous work found that SRI was overexpressed in ovarian cancer and the expression was much higher in chemoresistant ovarian cancer [19]. However, the expression of SRI in other cancers was unclear. Here, we further examined the SRI expression in multiple cancers using a human TissueArray. We found that SRI was overexpressed in most cancer tissues compared with their corresponding para-tumor tissues and normal tissues (Fig. 1a; Additional file 1: Figure S1). The immunohistochemistry (IHC) was scored from 0 (negative staining), 1 (weak-positive staining), 2 (moderate-positive staining), to 3 (strong-positive staining). In malignant tumors, the majority of scores was 2–3 compared with para-tumor tissues, and in normal tissues, the majority of scores was 1 (Fig. 1b). Through statistical analysis of the expression of SRI protein in different cancers, we found that, except for liver cancer, SRI expression was significantly higher in malignant tumor tissues than that in the para-tumor tissues (Fig. 1c), indicating that SRI is a tissue biomarker for most solid cancers.

### The expression of sorcin is negatively correlated with the concentration of calcium ions in chemoresistant and sensitive cancer cells

**Fig. 2:**
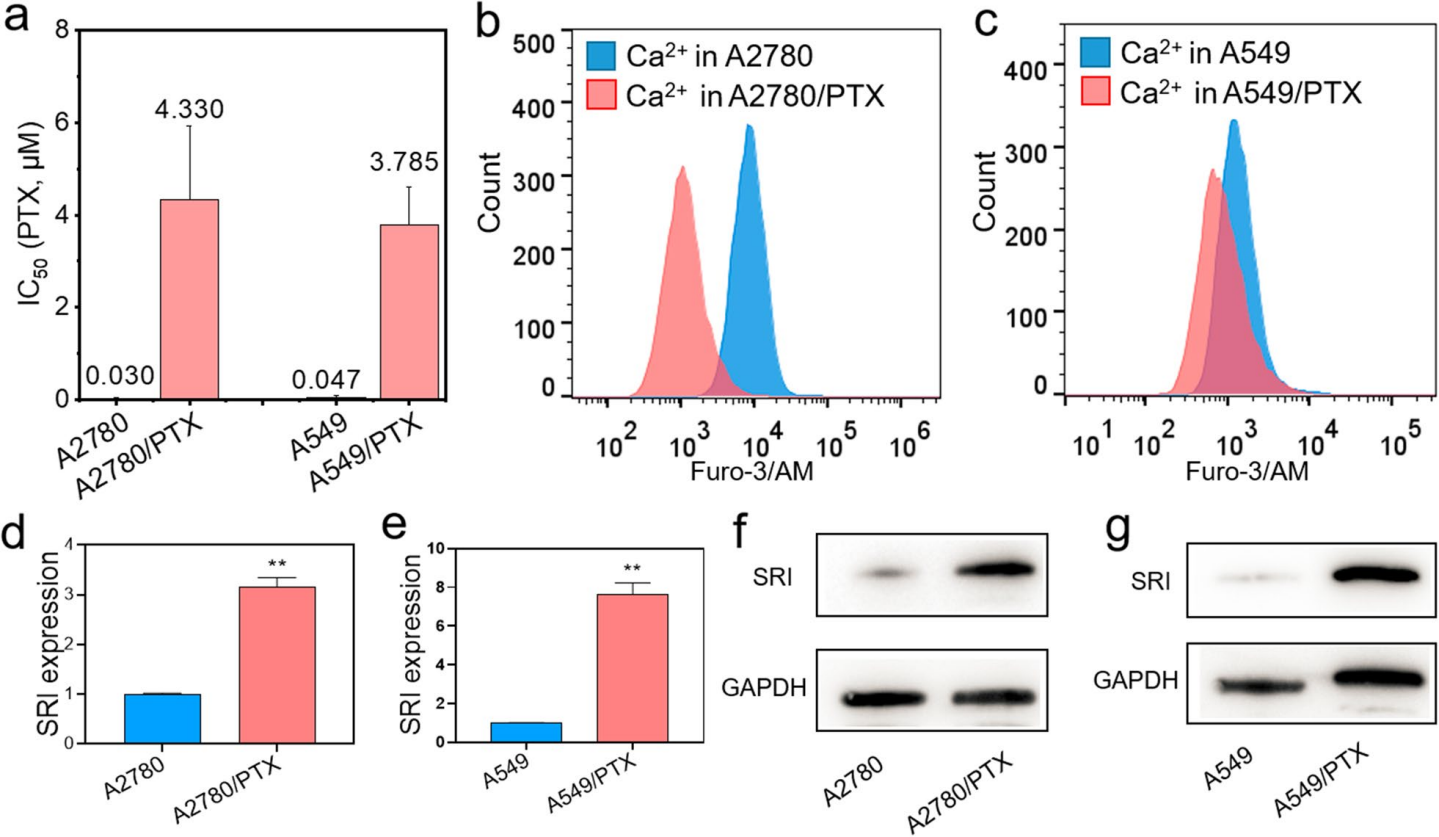
Calcium-related gene expression and calcium ion concentration in chemoresistant A2780/PTX and A549/PTX cells and sensitive counterparts A2780 and A549 cells. **a** Half-maximal inhibitory concentration (IC_50_) was measured in A2780/PTX and A549/PTX and their sensitive counterparts. **b–c** Calcium ion concentration was determined in A2780/PTX and A549/PTX and their sensitive counterparts by flow cytometry. **d–e** The expression of SRI mRNA was higher in A2780/PTX and A549/PTX compared with their sensitive counterparts determined by qRT-PCR. **f–g** The expression of SRI protein was detected in A2780/PTX and A549/PTX and their sensitive counterparts by Western blot. PTX, paclitaxel; SRI, sorcin. **P < 0.01

A2780/PTX (ovarian cancer) and A549/PTX (lung cancer) cells were chemoresistant, as ABCB1 was overexpressed at mRNA and protein levels in PTX-resistant cells detected by qRT-PCR and Western blot (Additional file 1: Figure. S2a, b, S3a, b) compared with their sensitive counterparts A2780 and A549 cells. Further, we confirmed that the half-maximal inhibitory concentration (IC_50_) was higher in A2780/PTX and A549/PTX cells than in their A2780 and A549 cells (Fig. 2a). Next, we found that the calcium ions level was lower in A2780/PTX and A549/PTX than in their sensitive counterparts detected by flow cytometry using Furo-3/AM fluorescent probe (Fig. 2b, c). In addition, we also found the overexpression of calcium-binding protein SRI at mRNA and protein levels in PTX-resistant cells detected by qRT-PCR (Fig. 2d, e) and Western blot (Fig. 2f, g). Thus, a high expression of SRI may be inevitably related to the reduction of intracellular calcium ion levels in chemoresistant cells. Clinically, SRI was highly expressed in ovarian and lung cancers (Additional file 1: Figure S4, S5) and patients with high expression of SRI had low overall survival (OS; P < 0.001) and progression-free survival (PFS; P = 0.003) in ovarian cancer (Additional file 1: Figure S6a, b) and PFS (P = 0.047) in lung cancer (Additional file 1: Figure S7b). Thus, we speculated that if the reduction of SRI expression in chemoresistant cancer cells, the calcium ion concentration may restore in the cytoplasm which may reverse the drug resistance.

### Dual-target nano-carrier LANP-PTX-siSRI is generated and characterized

**Fig. 3:**
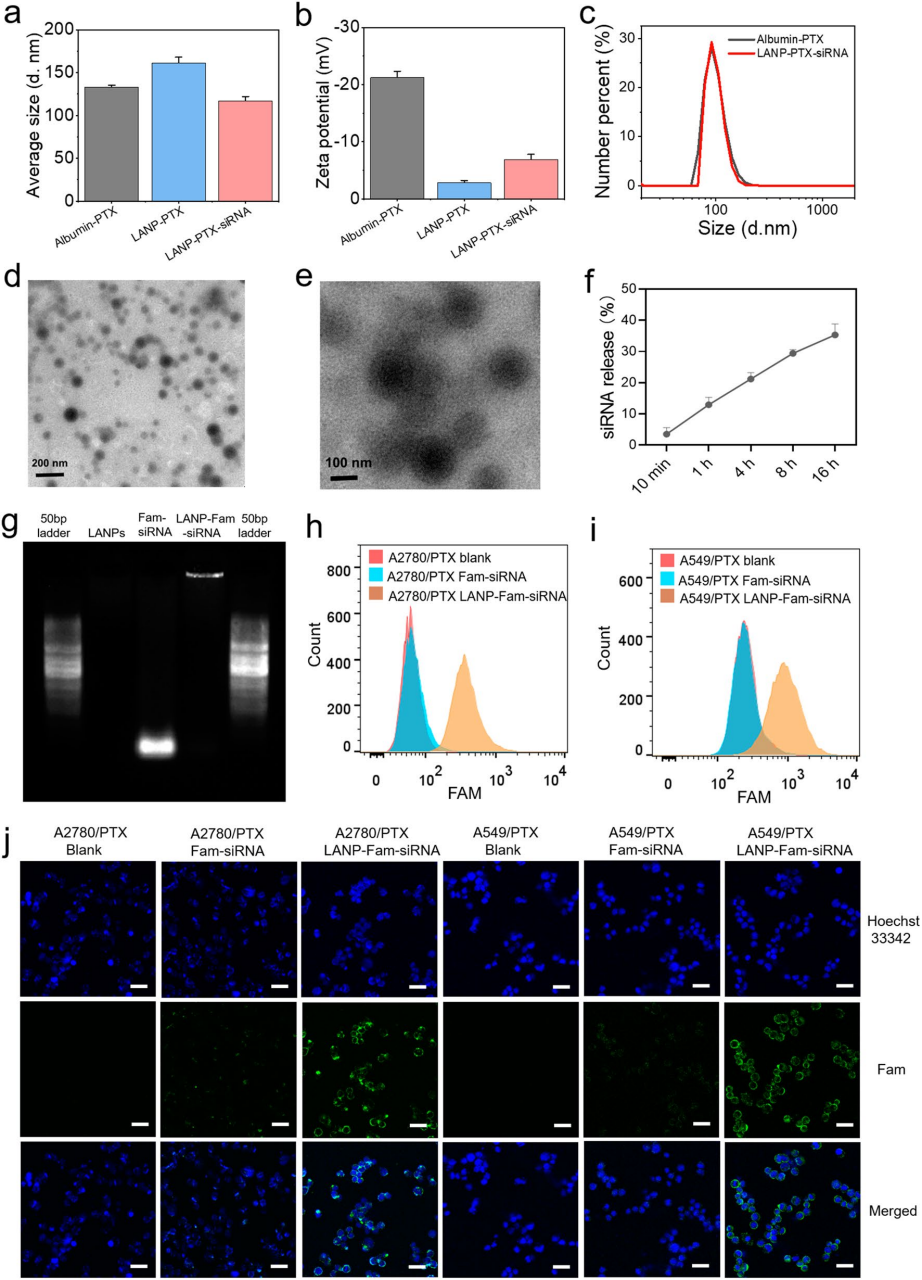
Characterization of lipid-coated albumin nanoparticles (LANP) and detection of siRNA-loaded LANP transfection efficiency. **a** The average size of albumin-PTX, lipid-coated albumin nanoparticles with PTX (LANP-PTX) and with PTX/SRI-siRNA (LANP-PTX-siSRI) was detected. **b** The Zeta potential of albumin-PTX, LANP-PTX, and LANP-PTX-siSRI was measured. **c** The particle size distribution of albumin-PTX and LANP-PTX-siSRI was analyzed. **d–e** LANP-PTX was photographed by the TEM. **f** The release of siRNA from LANP-PTX-siSRI into saline was measured. **g** Migration of blank LANP, Fam-labeled siRNA (Fam-siRNA), and LANP with Fam-labeled siRNA (LANP-Fam-siRNA) in agarose gel electrophoresis. **h–i** The Fam signals in A2780/PTX and A549/PTX were detected by flow cytometry. **j** The intracellular distribution of LANP-Fam-siRNA was detected by laser confocal microscopy. Scale bar, 25 μm

The inhibition of SRI expression may enhance intracellular calcium concentration and thus reverse the chemoresistance of cancer cells. To obtain a nano-carrier that can reduce SRI expression and increase the effectiveness of PTX in chemoresistant cancer cells, we designed a lipid-coated albumin-PTX nanoparticle that co-loaded PTX and SRI-siRNA (siSRI). Albumin-PTX is a nano-drug that has already been used in the clinic [28]. 1,2-dioleoyl-3-trimethyl-ammonium-propane (DOTAP) is a cationic lipid [29], which can bind electronegative siRNA via electrostatic force. Dioleoylphosphatidylethanolamine (DOPE) is an important helper lipid molecule [30], which can enhance transfection. Therefore, we prepared a lipid-coated albumin-PTX nanoparticle (LANP-PTX) by coating DOTAP and DOPE onto albumin-PTX nanoparticles. Then PTX/siSRI co-loaded albumin nanoparticles (LANP-PTX-siSRI) were obtained by mixing LANP-PTX with siSRI. By the characterization of the LANP-PTX and LANP-PTX-siSRI, we found the average particle size of the LANP-PTX and LANP-PTX-siSRI was at 100 ~ 150 nm (Fig. 3a), while the Zeta potential of LANP-PTX was significantly reduced after coated albumin-PTX by lipid, and the Zeta potential of LANP-PTX-siSRI was slightly reversed after the binding of siSRI (Fig. 3b), proving that the LANP can effectively bind siRNA through electrostatic force. The effect of zeta potential was an unavoidable problem for cationic lipid gene vectors because the vector loading capacity was dependent on the positively charged cationic lipids to bind to negatively charged nucleic acid. To reduce the influence of zeta potential, the cationic lipid content was then maintained at an appropriate level to enable the carrier to load siRNA while the zeta potential tended to zero. The distribution analysis showed that the size of LANP-PTX-siSRI was uniform (Fig. 3c). The transmission electron microscope (TEM) demonstrated that the morphology of the LANP-PTX was regular and well dispersed (Fig. 3d). A ring membrane was observed in LANP-PTX by TEM (Fig. 3e), indicating that the lipid was self-assembled onto the surfaces of the nanoparticles. The releasing ability of siRNA was detected by placing LANP-PTX-siSRI into a microdialysis tube and measuring the concentration of siRNA in dialysates. The results showed that the LANP-PTX-siSRI effectively released siRNA in normal saline (Fig. 3f). Because free siRNAs can migrate out from a well in agarose gel electrophoresis (AGE) but nanoparticle-bound siRNAs do not due to the loading of nanocarriers, we expected to observe a distinct pattern of migration. Indeed, ACE displayed that Fam-labeled siRNAs (Fam-siRNAs) sufficiently migrated as a bright band shown in the gel, while LANP with Fam-labeled siRNA (LANP-Fam-siRNA) did not (Fig. 3g). Next, we verified the transfection efficiency of LANP-Fam-siRNA in vitro. Because siRNAs were labeled with green fluorescent dye Fam, cells transfected with Fam-siRNAs and LANP-Fam-siRNA can be detected by flow cytometry. We found that LANP-Fam-siRNA was successfully transported into chemoresistant cancer cells (Fig. 3h, i). Confocal microscopic observation further confirmed that the LANP-Fam-siRNA-transfected cells appeared bright green (Fig. 3j), while cells transfected with Fam-siRNAs without nanoparticles showed no or weak green signals, indicating that LANP can effectively deliver siRNAs into PTX-resistant cancer cells.

### LANP-PTX-siSRI inhibits PTX-resistant cancer cells in vitro

**Fig. 4:**
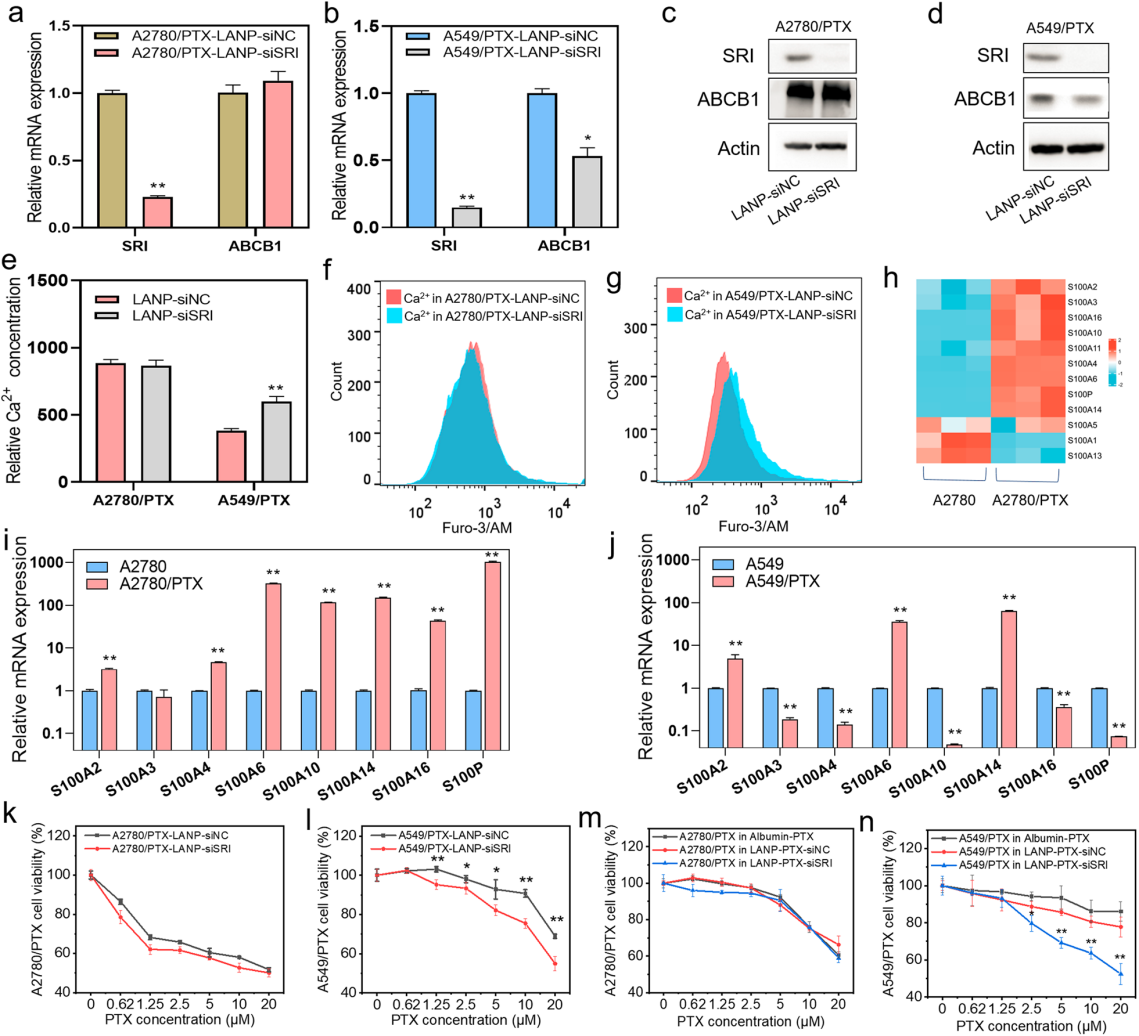
The effect of LANP-siSRI and LANP-PTX-siSRI in vitro. **a–b** Expression of SRI and ABCB1 mRNA in A2780/PTX and A549/PTX cells after the knockdown of SRI by LANP-siSRI determined by qRT-PCR. **c–d** Expression of SRI and ABCB1 protein in A2780/PTX and A549/PTX cells after the knockdown of SRI by LANP-siSRI detected by Western blot. **e** Measurement of calcium ion concentration in A2780/PTX and A549/PTX cells after the knockdown of SRI by LANP-siSRI detected by flow cytometry. **f–g** Flow cytometry data analysis of fluorescence intensity reflected calcium ion concentration in A2780/PTX (**f**) and A549/PTX (**g**) after the knockdown of SRI by LANP-siSRI. **h** Heatmap revealing the overexpression of S100 series genes in A2780/PTX detected by mRNA sequencing. **i-j** Expression of S100 series genes in A2780/PTX (**i**) and A549/PTX (**j**) detected by qRT-PCR. **k–l** Cell viability detection in A2780/PTX (**k**) and A549/PTX (**l**) after the knockdown of SRI by LANP-siSRI. **m–n** Cell viability detection in A2780/PTX (**m**) and A549/PTX (**n**) after treatment of LANP-PTX-siSRI. *P < 0.05; **P < 0.01

Next, we examined the effect of siSRI and PTX in combination with siSRI in chemoresistant cancer cells. The expression of SRI was decreased at mRNA and protein levels in A2780/PTX and A549/TPX cells detected by qRT-PCR and Western blot after transfection of LANP-siSRI (Fig. 4a–d). It has been shown in the previous report that ABCB1 was synchronized with the SRI down-regulation [31]. The current study also showed that ABCB1 expression at mRNA and protein levels was significantly decreased in A549/PTX cells (Fig. 4b and d) but almost did not change in A2780/PTX cells (Fig. 4a and c) after SRI knockdown. Furthermore, we found that the concentration of calcium ions did not change in A2780/PTX but significantly increased in A549/PTX after SRI knockdown (Fig. 4e–g). To figure out the difference between these two types of cells, we performed mRNA sequencing and focused on calcium-associated genes. The heatmap showed that the S100 series genes were overexpressed in A2780/PTX cells compared with A2780 cells (Fig. 4h). S100 belongs to a calcium-binding protein family, which plays an important biological role in vivo by interacting with calcium ions [32, 33]. After a comparison of the expression of S100 series genes between PTX-resistant cells and PTX-sensitive cells, we found that most S100 series genes were overexpressed in A2780/PTX compared with its sensitive counterpart (Fig. 4i), while the difference was not significant between A549/PTX and A549 (Fig. 4j). Therefore, due to the overexpression of S100 series genes in A2780/PTX cells, only knockdown of SRI may not be enough to change the calcium ion concentration. To examine the effect of LANP-siSRI and LANP-PTX-siSRI on cell viability after knocking down of SRI, we performed the CCK-8 assay. Again, there was no difference in cell viability between LANP-siSRI and negative control (LANP-siNC) groups after A2780/PTX cells were exposed to PTX (Fig. 4k). However, in A549/PTX cells, the cell viability was significantly decreased after transfection of LANP-siSRI compared with LANP-siNC (Fig. 4l). After dual-targets by siSRI and PTX, there was no difference in A2780/PTX cell viability between LANP-PTX-siNC and LANP-PTX-siSRI groups (Fig. 4m). Again in A549/PTX, the cell growth was significantly inhibited by dual-targeted LANP-PTX-siSRI (Fig. 4n). All these data suggest that LANP-PTX-siSRI can reverse chemoresistance in A549/PTX cells rather than in A2780/PTX cells because the knocking down of SRI can decrease the expression of ABCB1 and S100 genes such as A3, A4, A10, A16, and P, leading to an increase in intracellular calcium ion levels.

### LANP-PTX-siSRI suppresses PTX-resistant tumor growth in vivo

**Fig. 5:**
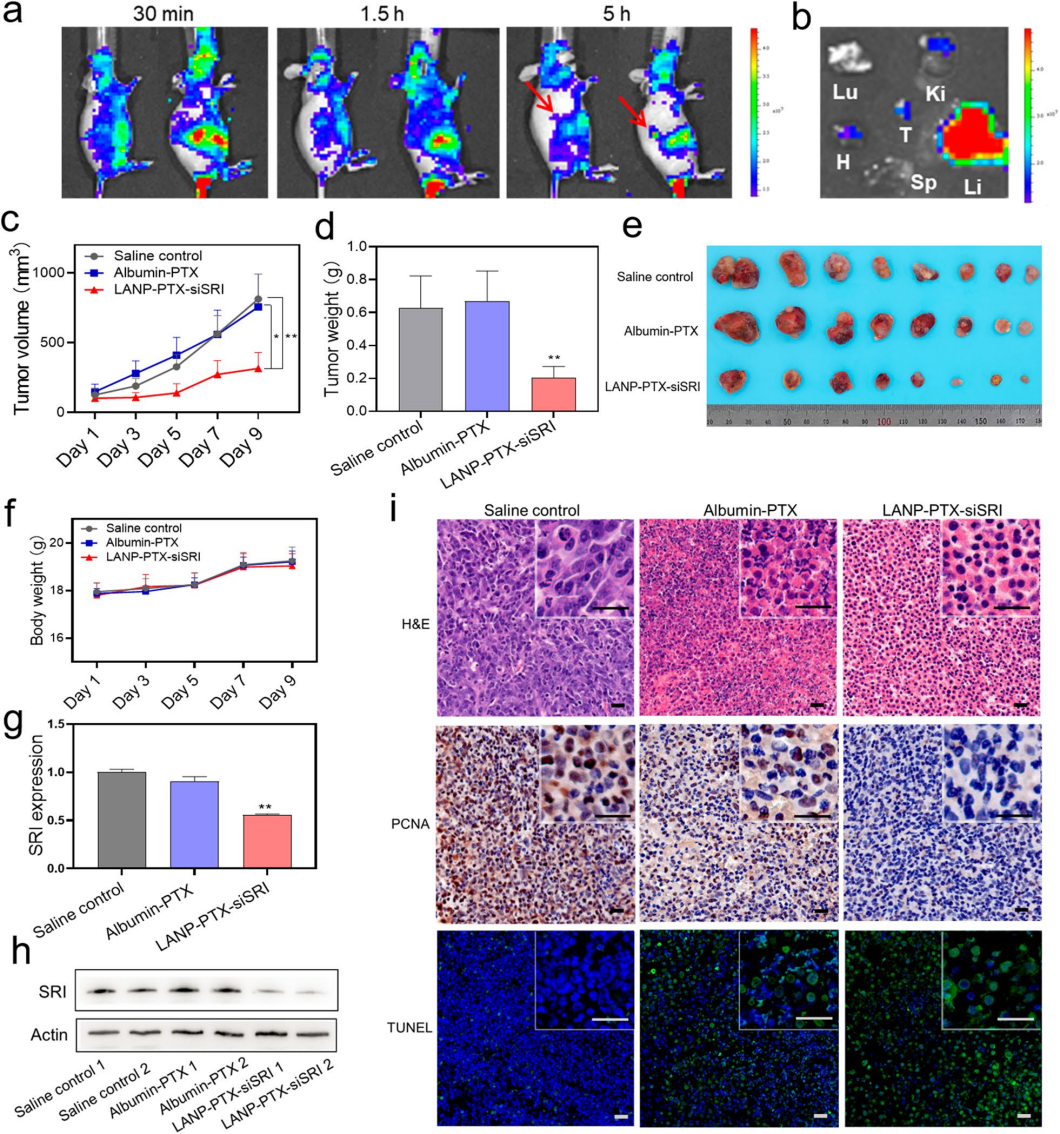
Suppression of PTX-resistant tumor growth by LANP-PTX-siSRI in vivo. **a** In vivo fluorescence imaging of Cy5.5 fluorescent-labeled LANP-PTX-siSRI (LANP-Cy5.5) at 30 min, 1.5 h, and 5 h after intravenous injection. Red arrows indicate the locations of tumors. **b** Ex vivo biodistributions of LANP-Cy5.5 at 5 h after intravenous injection. The column shows the intensity of ex vivo fluorescence and the major organs and the tumor were imaged. T, H, Li, Sp, Lu, and Ki stand for tumor, heart, liver, spleen, lung, and kidney, respectively. **c** Measurement of tumor volume during normal saline, albumin-PTX, and LANP-PTX-siSRI treatment. **d** Measurement of tumor weight in saline control, albumin-PTX, and LANP-PTX-siSRI groups after treatment. **e** Images of xenograft tumor after the sacrifice of mice. **f** Measurement of body weight in saline control, albumin-PTX, and LANP-PTX-siSRI groups during treatment. **g** Expression of SRI mRNA in tumor tissue of various groups after treatment determined by qRT-PCR. **h** Expression of SRI protein in tumor tissue of various groups after treatment detected by Western blot. **i** H&E, PCNA, and TUNEL stained tumor slices from various groups. Scale bar, 20 μm; *P < 0.05; **P < 0.01

It has been shown that nanoscale drugs can be enriched in the tumor by EPR effects [34], which can improve the therapeutic effect and reduce side effects [35]. To verify whether the LANP-PTX-siSRI improves the PTX effect on chemoresistant tumor growth in vivo, we conducted animal experiments. First of all, we generated Cy5.5 fluorescent-labeled LANP-PTX-siSRI (LANP-Cy5.5) and injected LANP-Cy5.5 into nude mice to evaluate the distribution of nanoparticles in mice. We found that LANP-Cy5.5 was enriched in the tumor at 5 h post-injection (Fig. 5a). The ex vivo biodistribution was observed in the tumor and main organs after the mice were sacrificed at 5 h (Fig. 5b), confirming the accumulation of the nanoparticles in the tumor. Because the liver is the main organ responsible for drug metabolism, most types of nanocarriers can be found to be accumulated and metabolized in the liver [36]. Next, we divided mice into three groups: normal saline group as a blank control, albumin-PTX group, and LANP-PTX-siSRI group. After 2 weeks of treatment with nano-drugs, the mice were sacrificed for further analyses. The growth of the tumor volume in various groups was tracked every 2-day during the treatment and the tumor was weighed and photographed after sacrifice. We found that the tumor growth rate in the LANP-PTX-siSRI group was significantly lower than that of the other two groups (Fig. 5c). Furthermore, the LANP-PTX-siSRI group also showed the lightest tumor weight and smallest tumor volume (Fig. 5d, e; Additional file 1: Figure S8), suggesting that the LANP-PTX-siSRI as a potential drug has a good inhibitory effect on chemoresistance in PTX-resistance cancers. In addition, there was no significant difference in body weight between groups during a period of treatment (Fig. 5f). These results further confirmed that A549/PTX was a PTX-resistant cell line with high resistance to PTX since a single target by albumin-PTX did not affect the tumor growth. Interestingly, the PTX-resistant tumor growth was significantly suppressed by dual-target using LANP-PTX-siSRI. To validate the effectiveness of knocking down SRI by LANP-PTX-siSRI, the expression of SRI in tumor tissues was examined. We found that SRI expression at mRNA and protein levels was significantly decreased in the LANP-PTX-siSRI group determined by qRT-PCR and Western blot, respectively, compared with saline control and albumin-PTX groups (Fig. 5g, h). Furthermore, H&E, PCNA, and TUNEL assays showed that more necrotic tissue, less proliferating cells, and more apoptotic cells were found in the LANP-PTX-siSRI group (Fig. 5i), indicating that LANP-PTX-siSRI had a good therapeutic effect on chemoresistant cancers. To verify the safety of LANP-PTX-siSRI, we examined the histological structure of tissues from the main organs of the mice including the heart, liver, spleen, lung, and kidney using H&E staining. The dosage of LANP-PTX-siSRI was calculated to be less than 2 mg/mL in the pilot experiment of an in vivo study. The hemolysis rate of LANP-PTX-siSRI was detected to be 0.75% ± 0.14% at a concentration of 2 mg/mL. We found that there was no serious impairment in the main organs (Additional file 1: Figure S9) because of a low hemolysis rate (Additional file 1: Figure S10), proving that the LANP-PTX-siSRI had good biological safety. All these results indicate that dual-target of the knockdown of SRI in combination with PTX by LANP-PTX-siSRI can achieve effective treatment for PTX-resistant cancer.

### TGF-β1 decreases SRI expression and increases intracellular calcium ion concentration

**Fig. 6:**
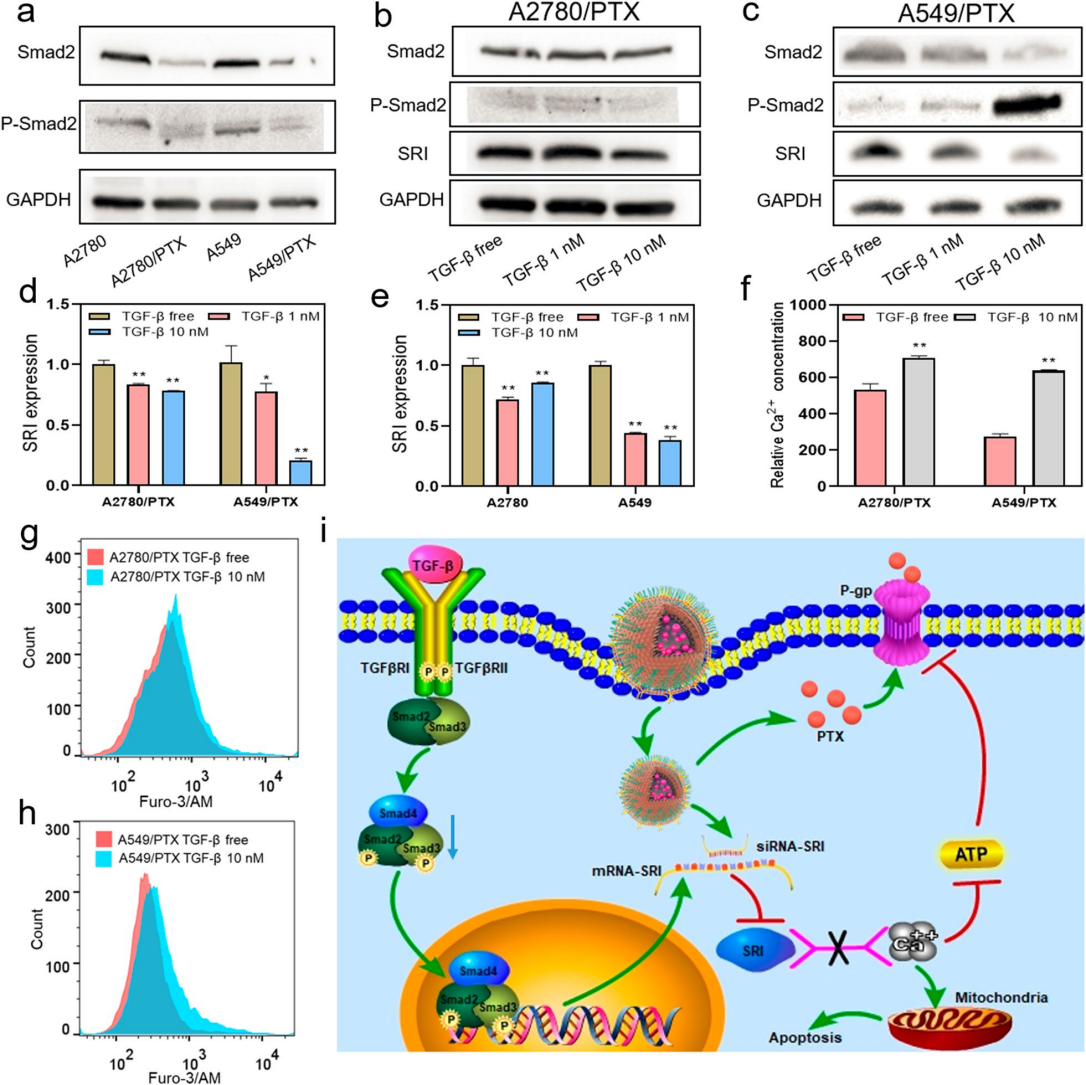
Effect of TGF-β signaling and LANP-PTX-siSRI in reversal cancer chemoresistance. **a** Expression of Smad2 and P-Smad2 in chemoresistant A2780/PTX and A549/PTX cells was detected by Western blot. **b–c** Expression of SRI protein in A2780/PTX and A549/PTX after TGF-β administration was detected by Western blot. **d–e** Expression of SRI mRNA after TGF-β administration was detected by qRT-PCR. **f** The concentration of calcium ions in A2780/PTX and A549/PTX after TGF-β administration was detected by flow cytometry. **g–h** Fluorescence intensity reflected calcium ion concentration in A2780/PTX (**g**) and A549/PTX (**h**) after TGF-β administration was detected by flow cytometry. **i** Schematic illustration of LANP-PTX-siSRI in reversal cancer chemoresistance

Our previous study demonstrated that SRI and transforming growth factor-β (TGF-β) are mutually regulated in ovarian cancer cells [19]. TGF-β is a cytokine that regulates cell growth and differentiation and has bidirectionally roles in cancer cell growth and metastasis at the different stages of cancer development [37]. We speculated that TGF-β can influence SRI expression and calcium ion homeostasis in chemoresistant cancer cells, which may lead to the PTX-resistant cells re-sensitize to a chemo-drug. First, we examined the TGF-β signaling pathway in PTX-resistant cells and their counterpart cells by detecting total Smad2 and phosphorylated-Smad2 (P-Smad2), an active form of Smad2. Smad2 is a transducer protein of TGF-β signaling and was found to be downregulated in chemoresistant A2780/PTX and A549/PTX cells compared with A2780 and A549 cells (Fig. 6a). The administration of TGF-β1 led to an increase in P-Smad2 and a decrease in SRI expression (Fig. 6b–e, Additional file 1: Figure S11), along with the decrease of S100A14 expression (Additional file 1: Figure S12, S13). Moreover, the concentration of calcium ions was increased in PTX-resistant cells after 10 nM TGF-β1 administration (Fig. 6f, h). Through the current study, we proposed a mechanism of PTX-resistance as shown in Fig. 6i. During the process of chemoresistance, the TGF-β signaling pathway is impaired as TGF-β signaling transducer Smad2 is downregulated. The decrease of TGF-β signaling leads to an overexpression of SRI, which further decreases intracellular calcium ions. LANP-PTX-siSRI has dual-target roles in the regulation of SRI and the delivery of PTX into a chemo-resistant cell. After LANP-PTX-siSRI administration, PTX functions as a chemo-drug along with released siSRI intervenes SRI expression and restores the calcium balance in chemoresistant cancer cells, thus reversing chemoresistance.

## Conclusions

The current study develops a novel lipid-coated albumin-PTX nanoparticle that co-delivery PTX and SRI-siRNA, which has dual-target roles in the reversal of chemoresistance. PTX functions as a chemo-drug and siSRI functions as an inhibitor of the expression of SRI mRNA and in turn increases intracellular calcium concentration. The synergistic action of PTX and siSRI leads chemoresistant cancer cells to a decrease in viability, induction of apoptosis, and suppression of tumor growth in vitro and in vivo. Thus, LANP-PTX-siSRI has a potentially therapeutical application. In addition, in cancer cells with high expression of S100 genes, the reduction of SRI can not increase the concentration of calcium ions in the cytosol, and therefore, cannot reverse chemoresistance. Furthermore, the TGF-β signaling pathway is found to play an important role in the regulation of SRI expression in chemoresistant cancer cells.

## Supplementary Information

**Additional file 1:** **Table S1. **PCR Primer sequences. **Table S2. **siRNA sequences. **Figure S1. **Representative images of SRI protein expression in human malignant tumor tissues and para-tumor tissues by immunohistochemistry. Scale bar, 50 μm. **Figure S2. **The expression of ABCB1 mRNA was higher in A2780/PTX than in A2780 determined by qRT-PCR (**a**) and Western blot (**b**). ABCB1, ATP binding cassette subfamily B member 1. **Figure S3.** The expression of ABCB1 protein was detected in A549/PTX and its sensitive counterparts by qRT-PCR (**a**) and Western blot (**b**). **Figure S4. **The transcription level of SRI in serous ovarian cancer. Normal (n=88) vs malignant tumor (n=427). The data source is TCGA_GTEx (https://xenabrowser.net/datapages/). **Figure S5.** The transcription level of SRI in lung adenocarcinoma. Normal (n=347) vs malignant tumor (n=515). The data source is TCGA_GTEx (https://xenabrowser.net/datapages/). **Figure S6. **Kaplan–Meier survival curves of overall survival (OS, **a**) and progression-free survival (PFS, **b**) comparing the high and low expressions of SRI in serous ovarian cancer. **Figure S7. **Kaplan-Meier survival curves of OS (**a**) and PFS (**b**) comparing the high and low expressions of SRI in lung adenocarcinoma. **Figure S8. **Pictures of tumors from various groups after treatment. **Figure S9. **Images of H&E stained tissues of the heart, liver, spleen, lung, and kidney tumor of mice after normal saline, albumin-PTX, and LANP-PTX-siSRI treatment. Scale bar, 50 μm. **Figure S10. **Hemolysis rate of LANP-PTX-siSRI. **Figure S11. **Expression of SRI protein in A2780 after TGF-β administration detected by Western blot. **Figure S12. **Expression of S100A14 mRNA in A2780/PTX and A549/PTX after TGF-β intervention determined by qRT-PCR. **Figure S13. **Expression of S100A14 mRNA in A2780 and A549 after TGF-β intervention determined by qRT-PCR.

## Data Availability

The authors declare that the main data supporting the findings of this study are available within the article and the enclosed Supplementary Information files. Extra data are available from the corresponding author upon request.

## Abbreviations

ABCB1: ATP binding cassette subfamily B member 1
Ca^++^: Calcium ions
DOPE: Dioleoylphosphatidylethanolamine
DOTAP: 1,2-Dioleoyl-3-trimethyl-ammonium-propane
EPR: Enhanced permeability and retention
Fam-siRNAs: Fam-labeled siRNAs
LANP: Lipid-coated albumin nanoparticle
LANP-Cy5.5: Cy5.5 fluorescent-labeled LANP-PTX-siSRI
LANP-PTX: Lipid-coated albumin-PTX nanoparticle
LANP-PTX-siSRI: Lipid-coated albumin nanoparticle with PTX and SRI-siRNA
LANP-siNC: The negative control of LANP-siSRI
LANP-siSRI: SiSRI loaded LANP
LANP-PTX-siNC: The negative control of LANP-PTX-siSRI
P-gp: Glycoprotein
siSRI: SRI-siRNA
PTX: Paclitaxel
P-Smad2: Phosphorylated-Smad2
SRI: Sorcin
TGF-β: Transforming growth factor-β
