# Supplementary material for: Lipid-coated albumin-paclitaxel nanoparticles loaded with sorcin-siRNA reverse cancer chemoresistance via restoring intracellular calcium ion homeostasis
Source: J Nanobiotechnology. 2022 Jul 7;20:319. doi: 10.1186/s12951-022-01487-6 (PMC9264675; doi:10.1186/s12951-022-01487-6)
Supplement: Supplementary file 1 — Additional file 1: Table S1. PCR Primer sequences. Table S2. siRNA sequences. Figure S1. Representative images of SRI protein expression in human malignant tumor tissues and para-tumor tissues by immunohistochemistry. Scale bar, 50 μm. Figure S2. The expression of ABCB1 mRNA was higher in A2780/PTX than in A2780 determined by qRT-PCR (a) and Western blot (b). ABCB1, ATP binding cassette subfamily B member 1. Figure S3. The expression of ABCB1 protein was detected in A549/PTX and its sensitive counterparts by qRT-PCR (a) and Western blot (b). Figure S4. The transcription level of SRI in serous ovarian cancer. Normal (n=88) vs malignant tumor (n=427). The data source is TCGA_GTEx (https://xenabrowser.net/datapages/). Figure S5. The transcription level of SRI in lung adenocarcinoma. Normal (n=347) vs malignant tumor (n=515). The data source is TCGA_GTEx (https://xenabrowser.net/datapages/). Figure S6. Kaplan–Meier survival curves of overall survival (OS, a) and progression-free survival (PFS, b) comparing the high and low expressions of SRI in serous ovarian cancer. Figure S7. Kaplan-Meier survival curves of OS (a) and PFS (b) comparing the high and low expressions of SRI in lung adenocarcinoma. Figure S8. Pictures of tumors from various groups after treatment. Figure S9. Images of H&E stained tissues of the heart, liver, spleen, lung, and kidney tumor of mice after normal saline, albumin-PTX, and LANP-PTX-siSRI treatment. Scale bar, 50 μm. Figure S10. Hemolysis rate of LANP-PTX-siSRI. Figure S11. Expression of SRI protein in A2780 after TGF-β administration detected by Western blot. Figure S12. Expression of S100A14 mRNA in A2780/PTX and A549/PTX after TGF-β intervention determined by qRT-PCR. Figure S13. Expression of S100A14 mRNA in A2780 and A549 after TGF-β intervention determined by qRT-PCR. [file 12951_2022_1487_MOESM1_ESM.pdf]

Lipid-coated albumin-paclitaxel nanoparticles loaded with sorcin-siRNA reverse cancer chemoresistance via restoring intracellular calcium ion homeostasis

Chenglong Wang [1, ‡], Xiaolin Xu [1, ‡], Peipei Zhang [2], Shuhan Xiong [2], Jia Yuan [1], Xuzhu Gao [1], Wencai Guan [1], Fanchen Wang [1], Xin Li [1], Hongjing Dou [2, *], Guoxiong Xu [1, *]

[1] *Research Center for Clinical Medicine, Jinshan Hospital, Fudan University, Shanghai 201508, P. R. China.*

[2] *State Key Laboratory of Metal Matrix Composites, School of Materials Science and Engineering, Shanghai Jiao Tong University, Shanghai 200240, P. R. China.*

[‡] *These authors contributed equally to this work.*

* Corresponding authors.

E-mail addresses: guoxiong.xu@fudan.edu.cn (G. Xu), ORCID iD: 0000-0002-9074-8754 (G. Xu); hjdou@sjtu.edu.cn (H. Dou).

Table S1. PCR Primer sequences.

Name	Primer Direction	Sequence (5’ → 3’)	GenBank Accession #
ABCB1	Forward	CACATTTGGCAAAGCTGGAGA	NM_001348945.2
	Reverse	CATCATTGGCGAGCCTGGTA	
Sorcin	Forward	GGACAAACTCAGGATCCGCTGTA	NM_003130.4
	Reverse	GCCGGCAAGTCTCCAGGTTA	
GAPDH	Forward	GCACCGTCAAGGCTGAGAAC	NM_002046.7
	Reverse	TGGTGAAGACGCCAGTGGA	
Actin	Forward	TCATCACCATTGGCAATGAG	NM_001101.5
	Reverse	CACTGTGTTGGCGTACAGGT	

Table S2. siRNA sequences.

Name	Sequence (5’ → 3’)	Target position
Sorcin		
Sense	GCUGGAGACAACACUUUAUTT	nt 323-341
Antisense	AUAAAGUAUUAUCUCCAGCTT	
FAM-labeled Negative control		
Sense	UUCUCCGAACGUGUCACGUTT	Scramble
Antisense	ACGUGACACGUUCGGAGAATT	
Negative control		
Sense	UUCUCCGAACGUGUCACGUTT	Scramble
Antisense	ACGUGACACGUUCGGAGAATT	

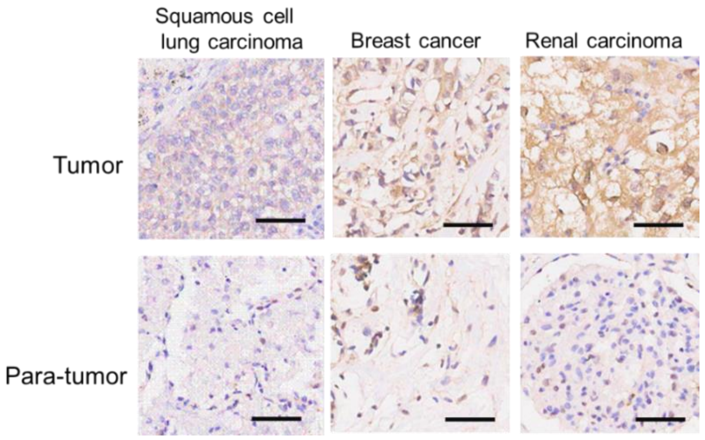

Figure S1. Representative images of SRI protein expression in human malignant tumor tissues and para-tumor tissues by immunohistochemistry. Scale bar, 50 μm.

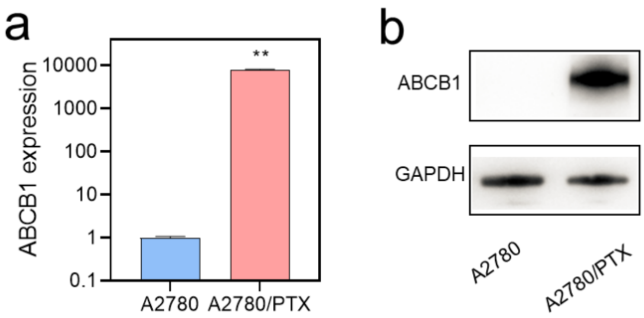

Figure S2. The expression of ABCB1 mRNA was higher in A2780/PTX than in A2780 determined by qRT-PCR (**a**) and Western blot (**b**). ABCB1, ATP binding cassette subfamily B member 1.

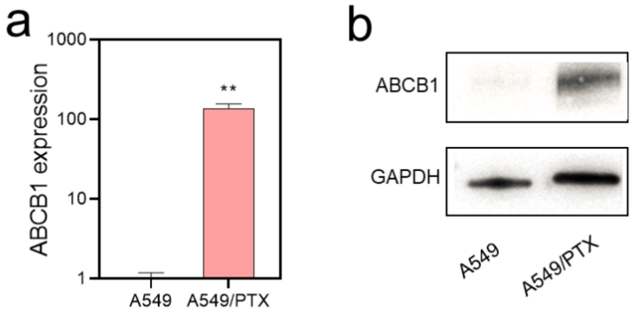

Figure S3. The expression of ABCB1 protein was detected in A549/PTX and its sensitive counterparts by qRT-PCR (**a**) and Western blot (**b**).

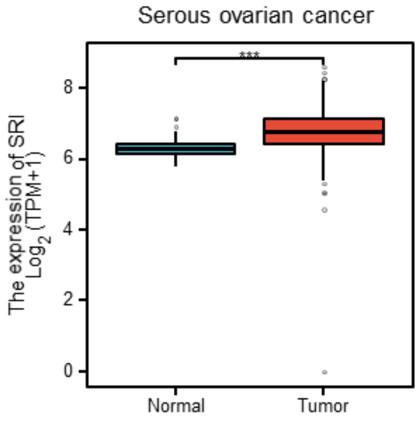

Figure S4. The transcription level of SRI in serous ovarian cancer. Normal (n=88) vs malignant tumor (n=427). The data source is TCGA_GTEx (https://xenabrowser.net/datapages/).

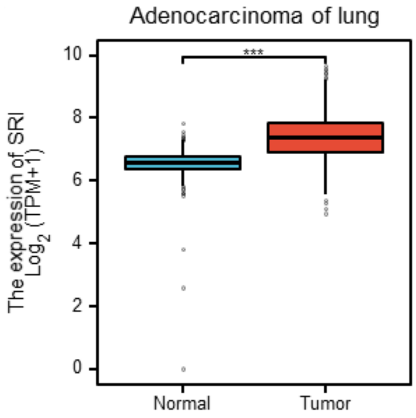

Figure S5. The transcription level of SRI in lung adenocarcinoma. Normal (n=347) vs malignant tumor (n=515). The data source is TCGA_GTEx (https://xenabrowser.net/datapages/).

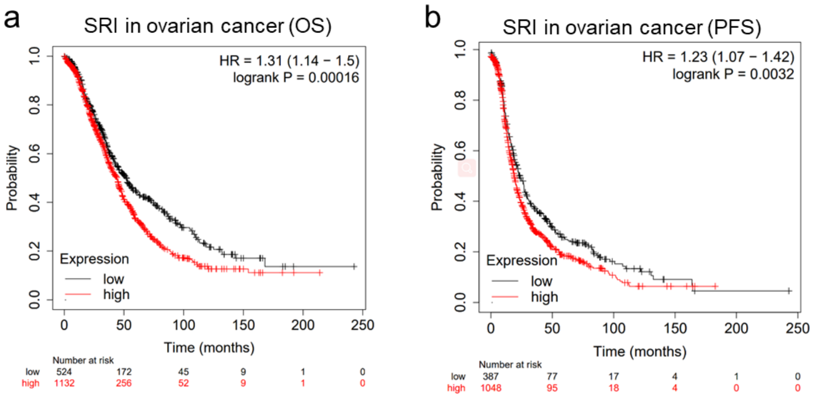

Figure S6. Kaplan–Meier survival curves of overall survival (OS, **a**) and progression-free survival (PFS, **b**) comparing the high and low expressions of SRI in serous ovarian cancer.

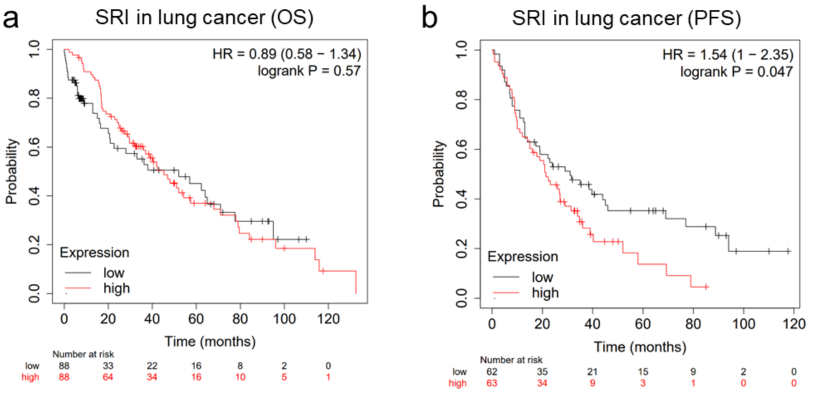

Figure S7. Kaplan-Meier survival curves of OS (**a**) and PFS (**b**) comparing the high and low expressions of SRI in lung adenocarcinoma.

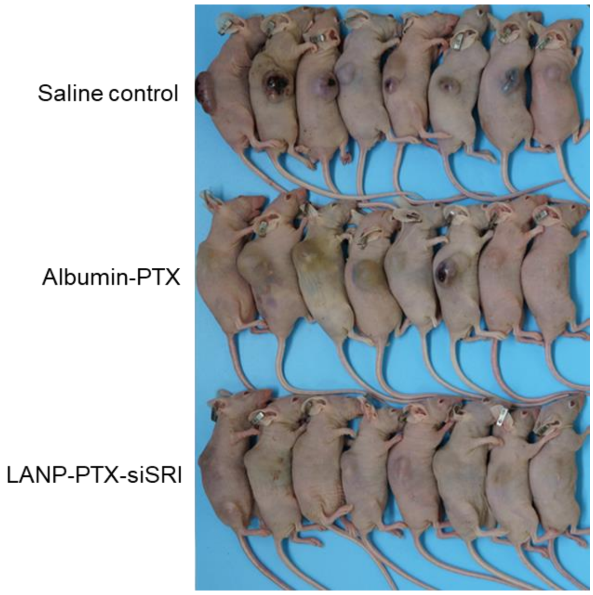

Figure S8. Pictures of tumors from various groups after treatment.

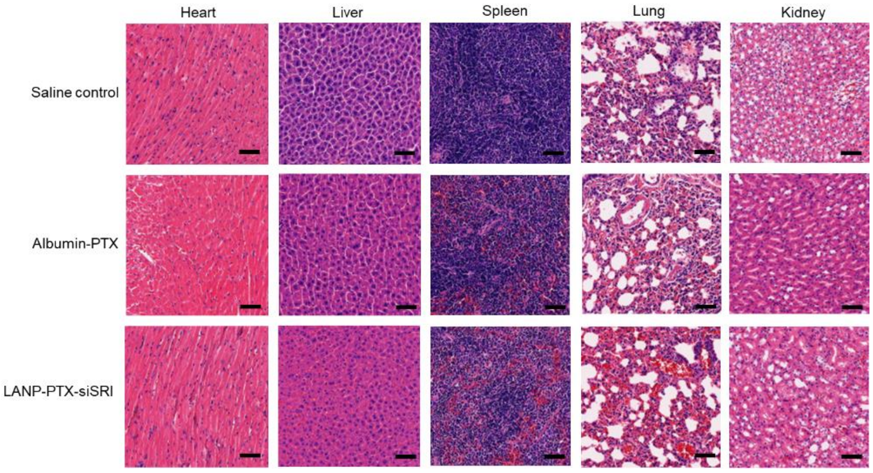

Figure S9. Images of H&E stained tissues of the heart, liver, spleen, lung, and kidney tumor of mice after normal saline, albumin-PTX, and LANP-PTX-siSRI treatment. Scale bar, 50 μm.

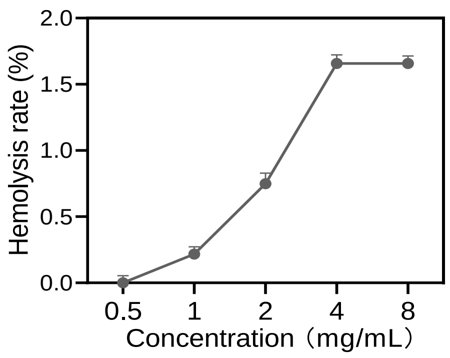

Figure S10. Hemolysis rate of LANP-PTX-siSRI.

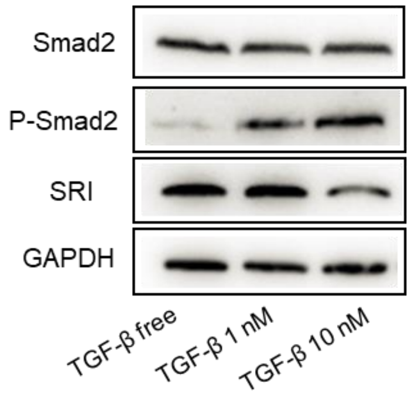

Figure S11. Expression of SRI protein in A2780 after TGF-β administration detected by Western blot.

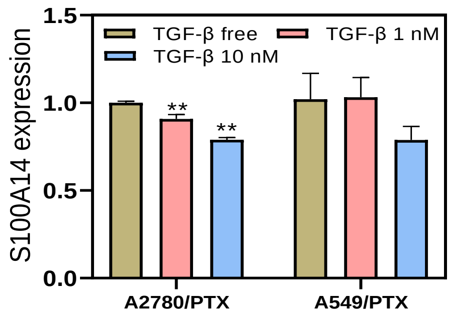

Figure S12. Expression of S100A14 mRNA in A2780/PTX and A549/PTX after TGF-β intervention determined by qRT-PCR.

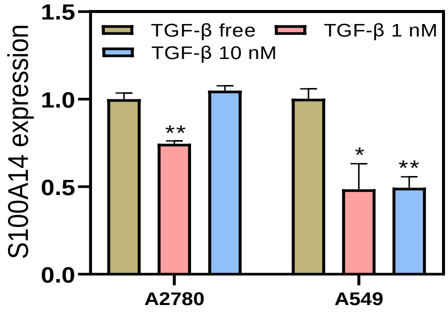

Figure S13. Expression of S100A14 mRNA in A2780 and A549 after TGF-β intervention determined by qRT-PCR.
